# Supplementary material for: Talin dissociates from RIAM and associates to vinculin sequentially in response to the actomyosin force
Source: Nat Commun. 2020 Jun 19;11:3116. doi: 10.1038/s41467-020-16922-1 (PMC7305319; doi:10.1038/s41467-020-16922-1)
Supplement: Supplementary file 1 — Supplementary Information [file 41467_2020_16922_MOESM1_ESM.pdf]

Talin dissociates from RIAM and associates to vinculin sequentially in response to the actomyosin

Vigouroux et al.

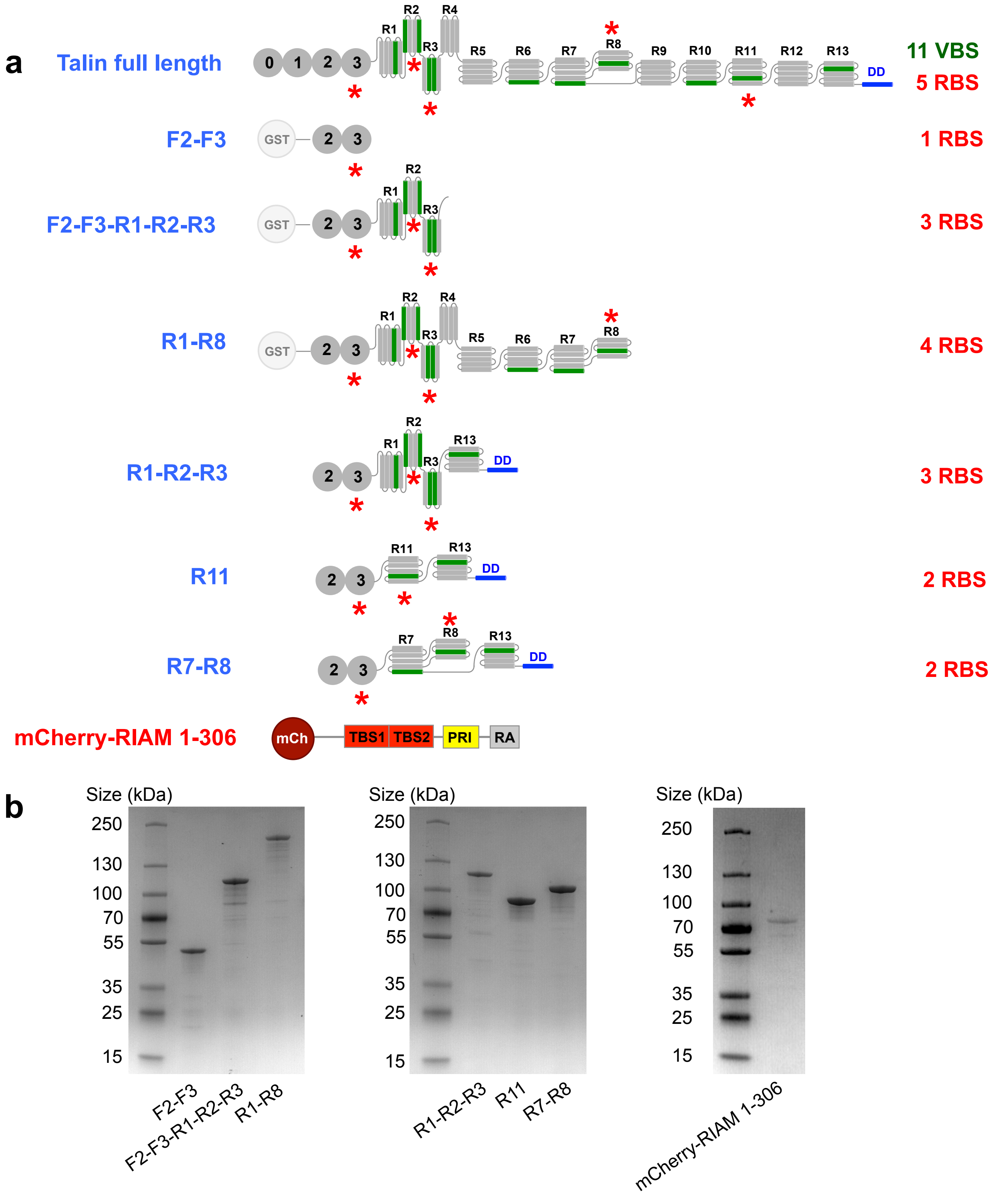

Supplementary Figure 1. Proteins used in the study. (a) Constructs used in this study. The talin domains named 0, 1, 2 and 3 are the F0, F1, F2 and F3 subdomains of the FERM (four-point-one, ezrin, radixin, moesin). R1 to R13 are the helix bundles along the rod domains of talin. DD is the dimerization domain of talin. Red stars show the RIAM binding sites (RBS) of talin. Dark green helices show the vinculin binding sites (VBSs) of talin. mCherry-RIAM 1-306 contains a N-terminal mCherry tag (mCh), two talin-binding site (TBS1 and TBS2), a Ras-association domain (RA) and a Plekstrin-homology domain (PH). **(b)** SDS-PAGE of the purified proteins used in this work, stained with Coomassie blue. Proteins were purified 3 times independently with similar results on SDS PAGE. Source data are provided as a Source Data file.

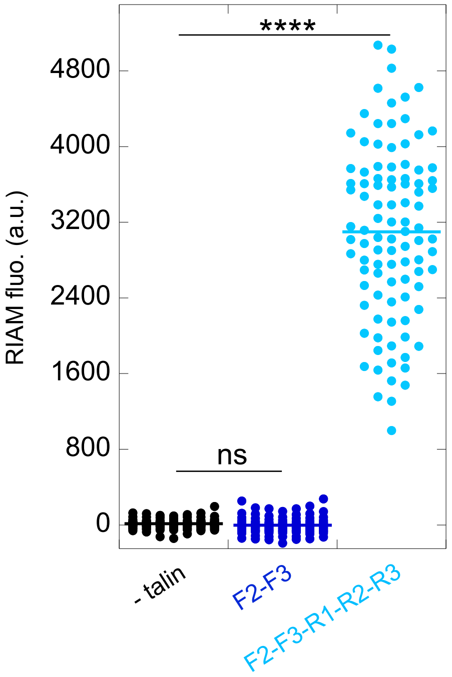

Supplementary Figure 2. The F3 subdomain of micropattern-bound talin does not bind to RIAM. Recruitment of RIAM 1-306 in non-coated disks and disks coated with talin F2-F3 or talin F2-F3-R1-R2-R3. Conditions: 1 µM mCherry-RIAM 1-306, 3 µM of each talin construct during the coating step. Each data point represents the mean fluorescence of a single disk. The bar shows the mean. n = 105, 105 and 98 disks for - talin, F2-F3 and F2-F3-R1-R2-R3 respectively. No significant difference (ns) was found between '– talin' and 'F2-F3' (P = 0.0940), a significant difference was found between '– talin' and 'F2-F3-R1-R2-R3' ($P = 1.69 \times 10^{-55}$) using a two-tailed t-test. ****, P<0.0001. Source data are provided as a Source Data file.

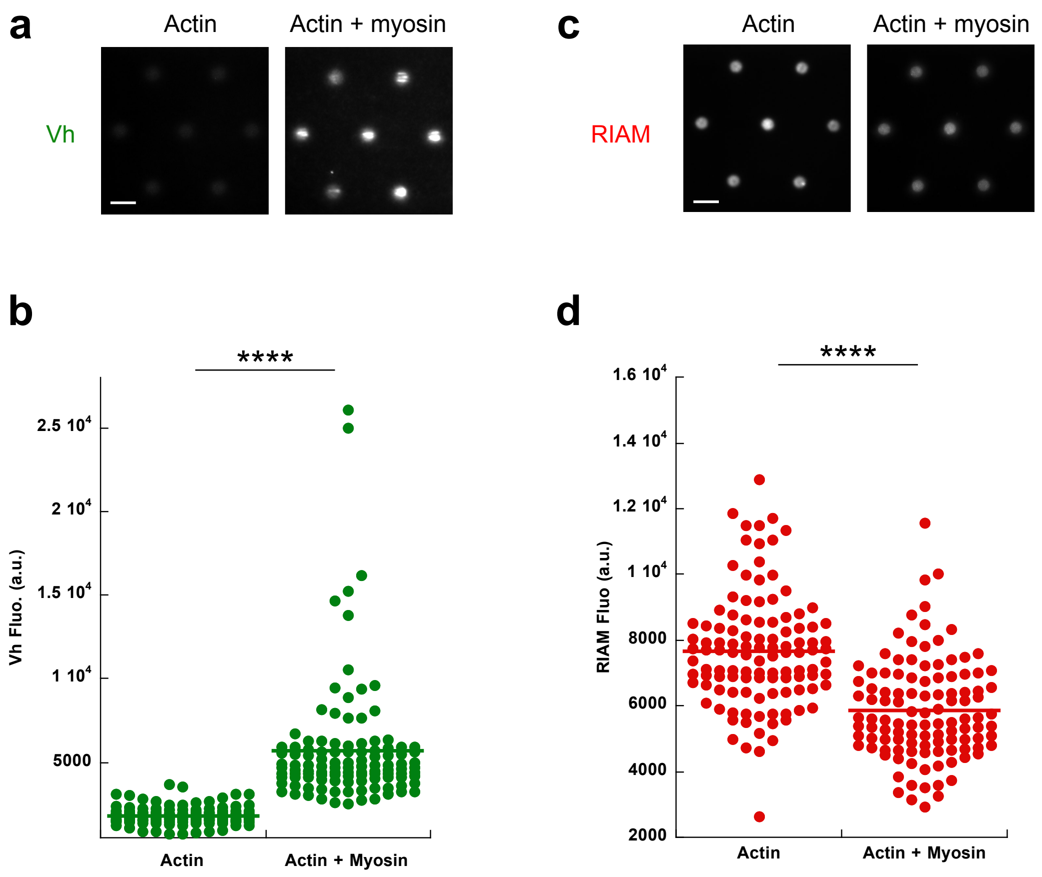

Supplementary Figure 3. Myosin II promotes the binding of Vh to talin R1-R2-R3 and the dissociation of RIAM from talin R1-R2-R3. (a, c) Representative images of the recruitment of Vh (a) or RIAM (c) in disks coated with talin R1-R2-R3 in the presence of short non polymerizing actin filaments alone (left) or with myosin II (right). Conditions: 100 nM EGFP-Vh (a) or 100 nM mCherry-RIAM 1-306 (c), 4.8 µM pre-formed gelsolin-capped short actin filaments, 25 nM myosin II, 1 µM of talin R1-R2-R3 during the coating step. Scale bar = 10 µm. **(b, d)** The recruitment of Vh (b) or RIAM (d) is quantified in the conditions described in (a) and (c) respectively. Each data point represents the mean fluorescence of a single disk, the bar shows the mean. n = 112 disks. A significant difference was found between 'Vh + actin' and 'Vh + actin + myosin' ($P = 4.28 \times 10^{-23}$) and between 'RIAM + actin' and 'RIAM + actin + myosin' ($P = 8.08 \times 10^{-15}$) using a two-tailed t-test. ****, P<0.0001. The experiment was repeated 4 times (a) and 3 times (c) independently with similar results. Source data are provided as a Source Data file.

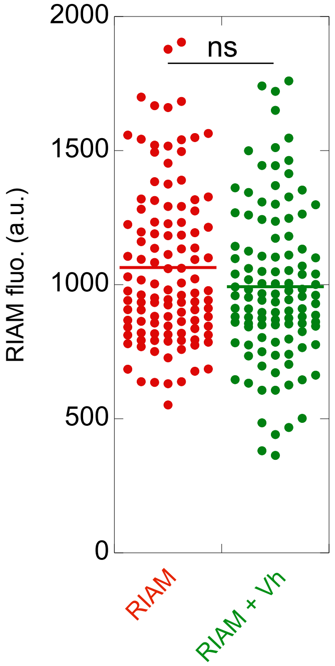

Supplementary Figure 4. Vh does not prevent RIAM binding to talin in the absence of actomyosin.

The recruitment of RIAM 1-306 is quantified in disks coated with talin R1-R2-R3 in the absence (left) and presence of Vh (right). Conditions: 100nM mCherry-RIAM 1-306, 500 nM EGFP-Vh and 1 μM of talin during the coating step. Each data point represents the mean fluorescence of a single disk. The bar shows the mean. n = 112 disks. No significant difference (ns) was found using a two-tailed t-test (P = 0.0677). Source data are provided as a Source Data file.

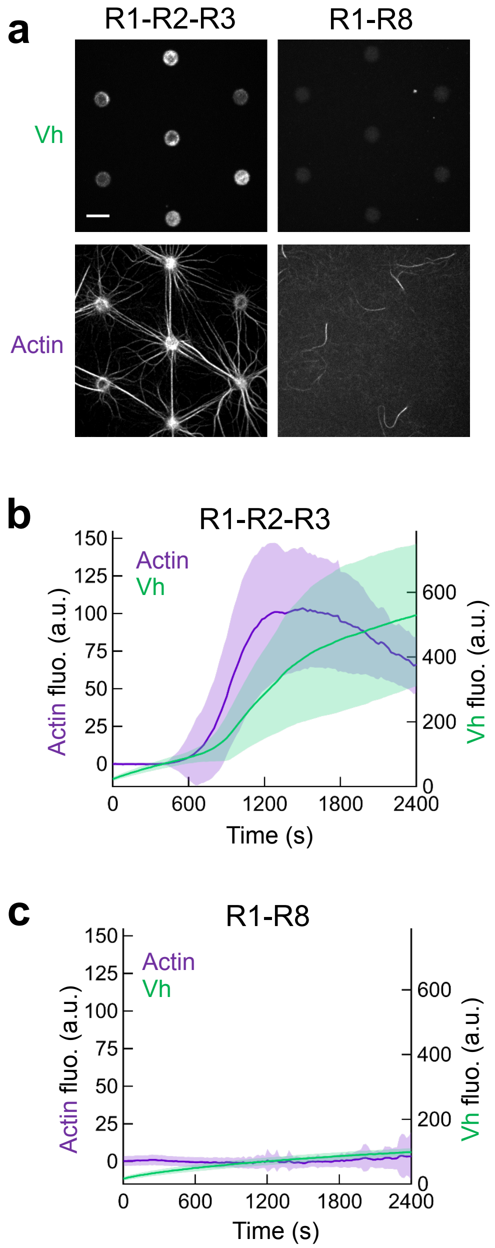

Supplementary Figure 5. The central actin-binding domain of talin (ABD2) is not capable of anchoring actin filaments to stretch talin and induce vinculin binding.

(a) Representative images of the recruitment of Vh (top) and actin (bottom) in disks coated with talin R1-R2-R3 (left) or talin R1-R8 (right) after 1000s. Talin R1-R2-R3 contains the C-terminal ABD3 (R13) whereas talin R1-R8 contains the same R1-R2-R3 domain followed by the central ABD2 (R4-R8). Conditions: 100 nM EGFP-Vh, 2.4 µM actin (1% Alexa647-labeled), 50 nM myosin, 1 µM of talin during the coating step. Scale bar = 10 µm. The experiment was repeated twice independently with similar results. **(b, c)** Average of the kinetics of EGFP-Vh and Alexa647-labeled actin presented in (a). Data are mean +/- SD. **(b)** n = 54 disks. **(c)** n = 48 disks. Source data are provided as a Source Data file.

Supplementary Table 1: Primer sequences for the constructs used in the study

Primer name	Primer description	Sequence (5' → 3')
Primer 1	talin R2-R3 forward	GGCGGTACCATTGGGGAAAGTGATACTGAC
Primer 2	talin R2-R3 reverse	GGCGGATCCACCGGATTTCTTGATGGCATTCTG
Primer 3	talin R13 forward	GGCGGATCCGGTGGAGACCCCACAGTCATTGCTG
Primer 4	talin R13-His6 reverse	GGCGAATTCTTAATGGTGATGGTGATGATGAC
Primer 5	talin F2-F3-R1-R2-R3 forward	GGCCGGTACCAAGTTCTTTTACTCAGACCAG
Primer 6	talin F2-F3-R1-R2-R3 reverse	GGCCCATGGAGCTAAAGATGTTC
Primer 7	talin F2-F3 (talin 196-405) forward<br>talin F2-F3-R1-R2-R3 (talin 196-911) forward<br>talin R1-R8 (talin 196-1659) forward	CGGGCGGATCCAAGTTCTTTTACTCAGACCAGAAT
Primer 8	talin F2-F3 (talin 196-405) reverse	CGGGCCTCGAGTCAGTGGTGGTGGTGGTGACCTCCGCT TTTTTTCTTCTTCAGGATGAT
Primer 9	talin F2-F3-R1-R2-R3 (talin 196-911) reverse	TTTTTGAATTCTCAATGGTGATGATGGTGGTGACCTCCTTTCT TGATGGCATTCTGCGCAGCTG
Primer 10	talin R1-R8 (talin 196-1659) reverse	CGCGAATTCTCAGTGGTGGTGGTGGTGACCTCCCAGCT GCCCTGGAGCCTTGTC
Primer 11	mCherry-RIAM 1-306 forward	GGCGGATCCATGGGTGAATCAAATGAAGAC
Primer 12	mCherry-RIAM 1-306 reverse	GGCCTCGAGCTAGTGATGGTGATGGTGATGGCCGCAGAAGC TTTCCTC
