# Supplementary material for: Talin dissociates from RIAM and associates to vinculin sequentially in response to the actomyosin force
Source: Nat Commun. 2020 Jun 19;11:3116. doi: 10.1038/s41467-020-16922-1 (PMC7305319; doi:10.1038/s41467-020-16922-1)
Supplement: Supplementary file 3 — Description of Additional Supplementary Files [file 41467_2020_16922_MOESM3_ESM.pdf]

Description of Additional Supplementary Files

Supplementary Movie 1. Talin R1-R2-R3 binds to vinculin in response to the actomyosin force. Movie showing the association of EGFP-Vh to disks coated with talin R1-R2-R3 in the absence (top) and presence of actomyosin (Vh is shown on the middle and actin on the bottom panel). Conditions: 100 nM EGFP-Vh, 2.4 µM actin (2% Alexa594-labeled), 50 nM myosin, 1 µM of talin R1-R2-R3 during the coating step. The images were acquired every 30s and color-coded using the fire LUT of ImageJ. AM, actomyosin.

Supplementary Movie 2. Talin R11 binds to vinculin in response to the actomyosin force. Movie showing the association of EGFP-Vh to disks coated with talin R11 in the absence (top) and presence of actomyosin (Vh is shown on the middle and actin on the bottom panel). Conditions: 100 nM EGFP-Vh, 2.4 µM actin (2% Alexa594-labeled), 50 nM myosin, 1 µM of R11 during the coating step. The images were acquired every 30s and color-coded using the fire LUT of ImageJ. AM, actomyosin.

Supplementary Movie 3. Talin R7-R8 does not bind to vinculin in response to the actomyosin force. Movie showing the association of EGFP-Vh to disks coated with talin R7-R8 in the absence (top) and presence of actomyosin (Vh is shown on the middle and actin on the bottom panel). Conditions: 100 nM EGFP-Vh, 2.4 µM actin (2% Alexa594-labeled), 50 nM myosin, 1 µM of R7-R8 during the coating step. The images were acquired every 30s and color-coded using the fire LUT of ImageJ. AM, actomyosin.

Supplementary Movie 4. The actomyosin force provokes the dissociation of RIAM from talin R1-R2-R3. Movie showing the dissociation of mCherry-RIAM 1-306 from disks coated with talin R1-R2-R3 in the absence (top) and presence of actomyosin (RIAM is shown on the middle and actin on the bottom panel). Conditions: 100 nM mCherry-RIAM 1-306, 2.4 µM actin (1% Alexa647-labeled), 50 nM myosin, 1 µM of talin R1-R2-R3 during the coating step. The images were acquired every 20s and color-coded using the fire LUT of ImageJ. AM, actomyosin.

Supplementary Movie 5. The actomyosin force provokes the dissociation of RIAM from talin R11. Movie showing the dissociation of mCherry-RIAM 1-306 from disks coated with talin R11 in the absence (top) and presence of actomyosin (RIAM is shown on the middle and actin on the bottom panel). Conditions: 100 nM mCherry-RIAM 1-306, 2.4 µM actin (2% Alexa488-labeled), 50 nM myosin, 1 µM of talin R11 during the coating step. The images were acquired every 60s and color-coded using the fire LUT of ImageJ. AM, actomyosin.

Supplementary Movie 6. The actomyosin force provokes a mild dissociation of RIAM from R7-R8. Movie showing the dissociation of mCherry-RIAM 1-306 from disks coated with talin R7-R8 in the absence (top) and presence of actomyosin (RIAM is shown on the middle and actin on the bottom panel). Conditions: 100 nM mCherry-RIAM 1-306, 2.4 µM actin (2% Alexa488-labeled), 50 nM myosin, 1 µM of talin R7-R8 during the coating step. The images were acquired every 60s and color-coded using the fire LUT of ImageJ. AM, actomyosin.

Supplementary Movie 7. Talin R1-R2-R3 switches from a RIAM-bound conformation to a vinculin-bound conformation in response to the actomyosin force. Movie showing (from left to right) the concomitant dissociation of RIAM 1-306, the association of Vh, the accumulation of actomyosin and a merge of Vh and RIAM in the same disks coated with talin R1-R2-R3. Conditions: 100 nM mCherry-RIAM, 100 nM EGFP-Vh, 2.4 µM actin (1% Alexa647-labeled), 50 nM myosin, 1 µM talin R1-R2-R3 during the coating step. The images were acquired every 20s and color-coded using the fire LUT of ImageJ. AM, actomyosin.

Supplementary Movie 8. Talin R11 switches from a RIAM-bound conformation to a vinculin-bound conformation in response to the actomyosin force. Movie showing (from left to right) the concomitant dissociation of RIAM 1-306, the association of Vh, the accumulation of actomyosin and a merge of Vh and RIAM in the same disks coated with talin R11. Conditions: 100 nM mCherry-RIAM, 100 nM EGFP-Vh, 2.4 µM actin (1% Alexa647-labeled), 50 nM myosin, 1 µM talin R11 during the coating step. The images were acquired every 60s and color-coded using the fire LUT of ImageJ. AM, actomyosin.

Supplementary Movie 9. RIAM inhibits the force-dependent binding of vinculin to talin R1-R2-R3. Movie showing the recruitment of Vh in disks coated with talin R1-R2-R3 in the absence of actomyosin (left), in the presence of actomyosin (middle) and in the presence of actomyosin and RIAM (right). Conditions: 100 nM EGFP-Vh, 2.4 µM actin, 50 nM myosin, 500 nM mCherry-RIAM, 1 µM talin R1-R2-R3 during the coating step. The images were acquired every 20s and color-coded using the fire LUT of ImageJ. AM, actomyosin.

Supplementary Movie 10. RIAM does not affect the force-dependent binding of vinculin to talin R11. Movie showing the recruitment of Vh in disks coated with talin R11 in the absence of actomyosin (left), in the presence of actomyosin (middle) and in the presence of actomyosin and RIAM (right). Conditions: 100 nM EGFP-Vh, 2.4 µM actin, 50 nM myosin, 3 µM mCherry-RIAM, 1 µM talin R11 during the coating step. The images were acquired every 60s and color-coded using the fire LUT of ImageJ. AM, actomyosin.
