# Supplementary material for: Talin dissociates from RIAM and associates to vinculin sequentially in response to the actomyosin force
Source: Nat Commun. 2020 Jun 19;11:3116. doi: 10.1038/s41467-020-16922-1 (PMC7305319; doi:10.1038/s41467-020-16922-1)
Supplement: Supplementary file 14 — Reporting Summary [file 41467_2020_16922_MOESM14_ESM.pdf]

nature research

Corresponding author(s): Christophe Le Clainche

Last updated by author(s): May 12, 2020

# Reporting Summary

Nature Research wishes to improve the reproducibility of the work that we publish. This form provides structure for consistency and transparency in reporting. For further information on Nature Research policies, see Authors & Referees and the Editorial Policy Checklist.

## Statistics

For all statistical analyses, confirm that the following items are present in the figure legend, table legend, main text, or Methods section.

| n/a | Confirmed | |
|---|---|---|
| ☐ | ☒ | The exact sample size ($n$) for each experimental group/condition, given as a discrete number and unit of measurement |
| ☐ | ☒ | A statement on whether measurements were taken from distinct samples or whether the same sample was measured repeatedly |
| ☐ | ☒ | The statistical test(s) used AND whether they are one- or two-sided<br>*Only common tests should be described solely by name; describe more complex techniques in the Methods section.* |
| ☒ | ☐ | A description of all covariates tested |
| ☒ | ☐ | A description of any assumptions or corrections, such as tests of normality and adjustment for multiple comparisons |
| ☐ | ☒ | A full description of the statistical parameters including central tendency (e.g. means) or other basic estimates (e.g. regression coefficient) AND variation (e.g. standard deviation) or associated estimates of uncertainty (e.g. confidence intervals) |
| ☐ | ☒ | For null hypothesis testing, the test statistic (e.g. $F$, $t$, $r$) with confidence intervals, effect sizes, degrees of freedom and $P$ value noted<br>*Give P values as exact values whenever suitable.* |
| ☒ | ☐ | For Bayesian analysis, information on the choice of priors and Markov chain Monte Carlo settings |
| ☒ | ☐ | For hierarchical and complex designs, identification of the appropriate level for tests and full reporting of outcomes |
| ☒ | ☐ | Estimates of effect sizes (e.g. Cohen's $d$, Pearson's $r$), indicating how they were calculated |

*Our web collection on statistics for biologists contains articles on many of the points above.*

## Software and code

Policy information about availability of computer code

| | |
|---|---|
| Data collection | MetaMorph 7.10 |
| Data analysis | ImageJ 1.51j8; IgorPro 8.0.4.2; KaleidaGraph 4.0; Excel 2016 |

For manuscripts utilizing custom algorithms or software that are central to the research but not yet described in published literature, software must be made available to editors/reviewers. We strongly encourage code deposition in a community repository (e.g. GitHub). See the Nature Research guidelines for submitting code & software for further information.

## Data

Policy information about availability of data

All manuscripts must include a data availability statement. This statement should provide the following information, where applicable:

- Accession codes, unique identifiers, or web links for publicly available datasets
- A list of figures that have associated raw data
- A description of any restrictions on data availability

Data supporting this manuscript are available from the corresponding author upon reasonable request. The source data underlying Figures 1c, 2e, 2f, 2g, 3e, 3f, 3g, 4d, 4e, 4f, 4g, 5d, 5e, 5f, 5g, Supplementary Figure 1b, 2, 3b, 3d, 4, 5b, 5c are provided as a Source Data file.

# Field-specific reporting

Please select the one below that is the best fit for your research. If you are not sure, read the appropriate sections before making your selection.

☒ Life sciences ☐ Behavioural & social sciences ☐ Ecological, evolutionary & environmental sciences

# Life sciences study design

All studies must disclose on these points even when the disclosure is negative.

| | |
|---|---|
| Sample size | The sample size n was defined as the number of disks quantified for one experiment in multiple fields of view. The sample size was not calculated. The sample size validity was based on our previous studies and prelimiary work. |
| Data exclusions | A very small number of aberrant fluorescence values were exceptionally excluded, with a maximum of 5% of all values in the exeriment. Exclusions were due to highly fluorescent microaggregates in Figure 3F, 3G. Exclusions were due to the malformation of a very small number of micropatterns, which does not allow actin anchoring to the disk, in Figure 2F, 2G, 5F and Supplementary Figure 5B. |
| Replication | Experiments were reproduced 2 to 11 times with the same results. Each figure pannel shows one representative experiment. |
| Randomization | Randomization is not relevant to our study. The experiments to be compared are performed the same day with the exact same materials. |
| Blinding | Investigators were not blinded because it is not necessary. The results of our experiments are not accessible during data acquisition. It only appears after quantification. Also, in most experiments, we don't expect particular results. Therefore, it is impossible for the person who is acquiring the data to influence the results during the experiments. |

# Reporting for specific materials, systems and methods

We require information from authors about some types of materials, experimental systems and methods used in many studies. Here, indicate whether each material, system or method listed is relevant to your study. If you are not sure if a list item applies to your research, read the appropriate section before selecting a response.

## Materials & experimental systems

| n/a | Involved in the study | |
|---|---|---|
| ☒ | ☐ | Antibodies |
| ☒ | ☐ | Eukaryotic cell lines |
| ☒ | ☐ | Palaeontology |
| ☒ | ☐ | Animals and other organisms |
| ☒ | ☐ | Human research participants |
| ☒ | ☐ | Clinical data |

## Methods

| n/a | Involved in the study | |
|---|---|---|
| ☒ | ☐ | ChIP-seq |
| ☒ | ☐ | Flow cytometry |
| ☒ | ☐ | MRI-based neuroimaging |
